# Molecular characteristics of *Staphylococcus aureus* isolates from food surveillance in southwest China

**DOI:** 10.1186/s12866-018-1239-z

**Published:** 2018-08-29

**Authors:** Feng Liao, Wenpeng Gu, Zushun Yang, Zhishuo Mo, Lu Fan, Yidan Guo, Xiaoqing Fu, Wen Xu, Chaoqun Li, Jiejie Dai

**Affiliations:** 1 Department of Respiratory Medicine, The First People’s Hospital of Yunnan province, Kunming, 650022 China; 2 The Affiliated Hospital of Kunming University of Science and Technology, Kunming, Yunnan China; 3 Center of Tree Shrew Germplasm Resources, Institute of Medical Biology, The Chinese Academy of Medical Science and Peking Union Medical College, Yunnan Key Laboratory of Vaccine Research and Development on Severe Infectious Diseases, Yunnan Innovation Team of Standardization and Application Research in Tree Shrew, Kunming, 650118 China; 4 Department of Acute Infectious Diseases Control and Prevention, Yunnan Provincial Centre for Disease Control and Prevention, Kunming, 650022 China; 5 Centre for Sanitary Inspection, Yunnan Provincial Centre for Disease Control and Prevention, Kunming, 650022 China; 6 The Third Affiliated Hospital of Sun Yat-Sen University, Guangzhou, 510630 China; 7 Wenshan Centre for Disease Control and Prevention, Wenshan, 663000 China; 8 Institute of Medical Biology, The Chinese Academy of Medical Science and Peking Union Medical College & Department of Acute Infectious Diseases Control and Prevention, Yunnan Provincial Centre for Disease Control and Prevention, Dongsi Street 158, Kunming, 650022 China; 9 Center of Tree Shrew Germplasm Resources, Institute of Medical Biology, Chinese Academy of Medical Sciences & Peking Union Medical School, Kunming, 650118 China

**Keywords:** *Staphylococcus aureus*, Food surveillance, Southwest China

## Abstract

**Background:**

Staphylococcal food poisoning (SFP) is one of the most common food-borne diseases in the world. Pulsed-field gel electrophoresis (PFGE), multilocus sequence typing (MLST) and *spa* typing methods were used to characterize *Staphylococcus aureus* isolates from food surveillance during 2013–2015 in southwest China, and Staphylococcal cassette chromosome *mec* (SCC*mec*) typing was used for methicillin-resistant *S. aureus* (MRSA). Isolates were also examined for their antibiotic resistance and carriage of virulence genes.

**Results:**

Isolation rate of *S. aureus* was 2.60% during the three years’ surveillance and 29.50% of them were MRSA. All the *S. aureus* had *hla* genes (100%), 14.34% of the strains had *tst*, and 16.73% had *PVL*. 163 PFGE-*SmaI* patterns, 41 ST types and 36 *spa* types were obtained for all the *S. aureus*. Among them, ST6-t701 (13.15%), ST7-t091 (12.75%), ST59-t437 (9.96%) and ST5-t002 (7.57%) were the prevalent genotypes. Most of MRSA in this study belonged to SCC*mec* IV and V, accounted for 74.32% and 20.27% respectively. ST6-SCC*mec* IV-t701 (36.50%) was the most prevalent clone among isolates from food, followed by ST59-SCC*mec* V-t437 (20.30%), ST5-SCC*mec* IV-t002 (12.20%) and ST59-SCC*mec* IV-t437 (12.20%). Some strains had the identical PFGE patterns, ST and *spa* types with isolates from patients.

**Conclusions:**

*S. aureus* isolated from food in southwest China displayed heterogeneity. Isolates had the same genotype profiles with isolates from patients, indicating high homology.

**Electronic supplementary material:**

The online version of this article (10.1186/s12866-018-1239-z) contains supplementary material, which is available to authorized users.

## Background

Food-borne diseases (FBD) represent major public health concern worldwide [1, 2]. Staphylococcal food poisoning (SFP) is one of the most common FBD in the world due to the ingestion of staphylococcal enterotoxins (SEs) that are produced by enterotoxigenic strains [3]. In the United States, SFP is the major concern of public health and it is one of the most common causes of reported FBD, causing an estimated 241,000 illnesses per year, imposing a great economic burden [4, 5]. In China, 371 outbreaks of bacterial FBDs have been reported from 2008 to 2010, involving 20,062 cases and 41 deaths [6]. Furthermore, 94 outbreaks of SFP were reported from 2003 to 2007 according to the National Monitoring Network with 2223 individual patients and 1186 hospitalizations. During these SFP outbreaks, *Staphylococcus aureus* was the fifth most isolated pathogenic bacteria followed by *Vibrio parahaemolyticus*, *Bacillus cereus*, *Bacillus proteus*, and *Salmonella* [7]. Since 2009, Food safety law of People’s Republic of China was enacted, and *S. aureus* was one of the bacteria that must be tested according to this law. Therefore, the systemic surveillance of *S. aureus* for food products is very important.

*S. aureus* is a commensal and opportunistic pathogen that can cause wide spectrum of infections, from superficial skin infections to severe and invasive disease [8]. The toxigenic *S. aureus* strains associated with SFP can express one or more of a family of genes coded for heat-stable enterotoxins. These genes showed high sequence homology in their structure and functions [1]. While, other virulence genes such as toxic shock syndrome toxin 1 (TSST-1), exfoliative toxins and cytolytic toxins (leukocidin and hemolysins) can also cause related diseases [9]. At present, the major molecular typing methods for *S. aureus* were pulsed-field gel electrophoresis (PFGE), multilocus sequence typing (MLST), *spa* typing and Staphylococcal cassette chromosome *mec* (SCC*mec*) typing [10–13]. PFGE was considered as the golden method for *S. aureus* typing, showing a high discriminatory power. MLST was suitable for analyzing the evolutionary relation and clone complex of the strains. SCC*mec* developed by International Working Group on the Classification of Staphylococcal Cassette Chromosome Elements, (IWG-SCC: www.sccmec.org) was mainly used for genotyping of MRSA [14].

Yunnan is located in southwest China, including 16 cities. The lifestyle and eating habits are specific, and there is no systemic surveillance program for *S. aureus* in foods at present. Therefore this study was the first analyzing the characteristics of *S. aureus* isolates from food surveillance between 2013 and 2015 in this province.

## Results

### Isolation of *S. aureus*

**Fig. 1:**
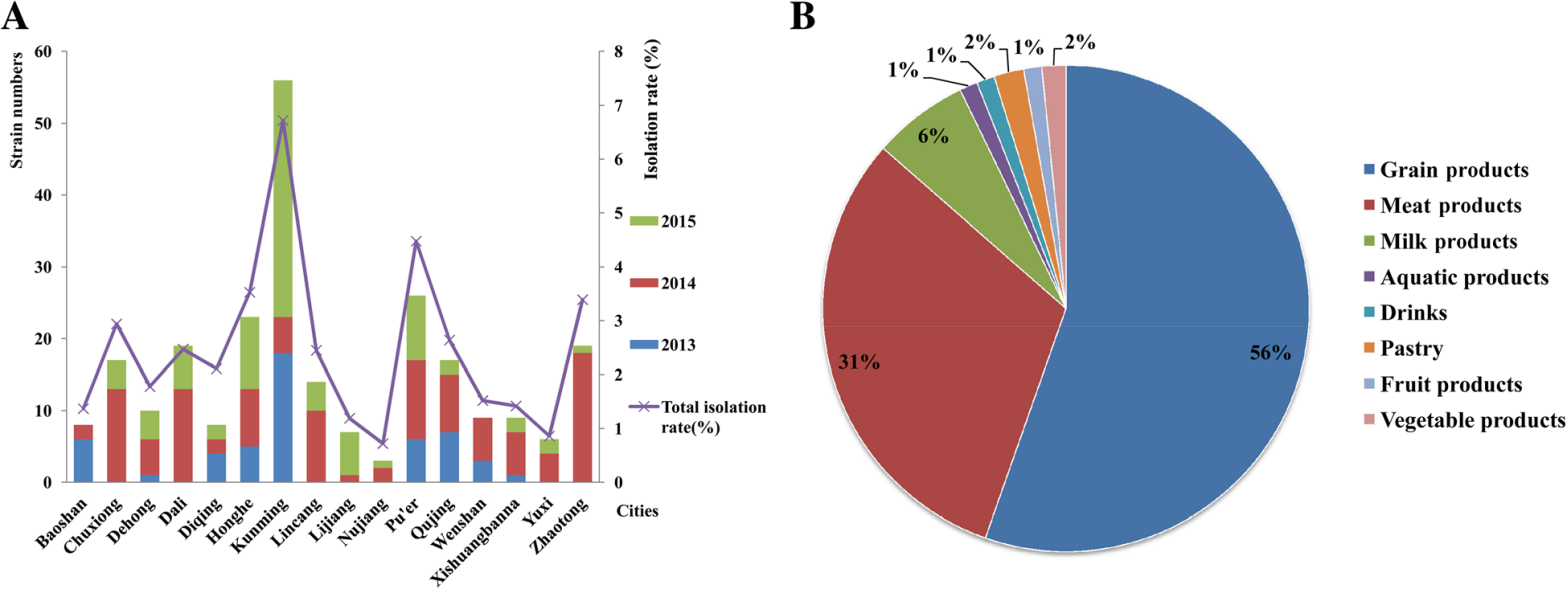
The isolation results of *S. aureus* in this study. **a**: The isolation rates and strain distributions; **b**: The isolation sources of the strains

The isolation rate of *S. aureus* was 2.60% (251/9646) for three years in Yunnan province. The highest isolation rate was 6.72% (56/833) in Kunming city followed by Pu’er 4.48% (26/581), Honghe 3.53% (23/651), Zhaotong 3.39% (19/561), Chuxiong 2.94% (17/579) and Qujing 2.64% (17/645). The lowest isolation rates were 0.72% (3/419) and 0.86% (6/699) observed in Nujiang and Yuxi respectively (Fig. 1a).

A total of 251 *S. aureus* strains were isolated from food, 56% (139/251) from grain products, 31% (78/251) from meat products and 6% (16/251) from dairy products, these strains accounted for 93% of all the isolates (Fig. 1b). The distribution of strains from different foods had statistical significance among the 16 cities (H = 39.55, *p* = 0.001).

### Antibiotic resistance

Seventy four *S. aureus* strains (29.50%) were MRSA exhibiting resistance to OXA, carrying the *mecA* gene. The resistance rate against to the other antibiotic was: 71.10% (180/251) to SXT, 51.00% (128/251) to EM, 46.60% (117/251) to TET, 44.20% (111/251) to CIP, and 37.80% (95/251) to CM. All isolates were sensitive to VAN (Table 1 and Additional file 1). Comparison of antibiotic resistance results between cities and sources, we found that only OXA (H = 30.52, *p* = 0.010) and GEN (H = 30.63, *p* = 0.010) had significance in 16 cities, while MXF (H = 29.46, *p* = 0.000) and TET (H = 33.63, *p* = 0.000) had statistical difference depending on food sources.

**Table 1:** The antibiotics resistant results in this study

Antibiotics	MICs	Strain numbers	Percent (%)
PCN	≤0.12	177	70.50%
	–	–	–
	≥0.25	74	29.50%
OXA	≤2	177	70.50%
	–	–	–
	≥4	74	29.50%
MXF	≤0.5	231	92.00%
	1	1	0.40%
	≥2	19	7.60%
SXT	≤2/38	71	28.30%
	–	–	–
	≥4/76	180	71.70%
LEV	≤1	78	31.10%
	2	155	61.80%
	≥4	18	7.20%
EM	≤0.5	118	47.00%
	1–4	5	2.00%
	≥8	128	51.00%
LZD	≤4	154	61.35%
	–	–	–
	≥8	97	38.65%
TET	≤4	134	53.40%
	8	0	0
	≥16	117	46.60%
CIP	≤1	8	3.20%
	2	132	52.60%
	≥4	111	44.20%
CM	≤0.5	133	53.00%
	1–2	23	9.20%
	≥4	95	37.80%
GEN	≤4	208	82.90%
	8	6	2.40%
	≥16	37	14.70%
RIF	≤1	248	98.80%
	2	0	0
	≥4	3	1.20%
VAN	≤2	251	100%
	4–8	0	0
	≥16	0	0

### Virulence genes

All the *S. aureus* in this study had *hla* genes, the positive rate of other enterotoxin genes were *sea* 30.86% (77/251), *seb* 49.00% (123/251), *sec* 17.28% (43/251), *sed* 57.77% (145/251), *seg* 19.12% (48/251), *seh* 11.55% (29/251), *sei* 34.26% (86/251), *sej* 15.54% (39/251), *sen* 14.34% (36/251), *sem* 17.93% (45/251) and *seo* 12.75% (32/251). Thirty six strains had toxic shock syndrome toxin gene *tst* (14.34%), and the positive rate of exfoliative toxin genes was 21.91% (55/251) for *eta* and 12.35% (31/251) for *etb* respectively. Forty two isolates (16.73%) had leucocytic toxin gene *lukS-PV-lukF-PV* (*PVL*), the positive rate of other virulence genes were *hlb* 96.30% (242/251), *hld* 90.12% (226/251), *hlg* 87.65% (220/251), *hlg-2* 85.19% (214/251) and *edin* 35.46% (89/251). The virulence genes results had no statistical significance with source or distributions of the bacteria.

### PFGE

**Fig. 2:**
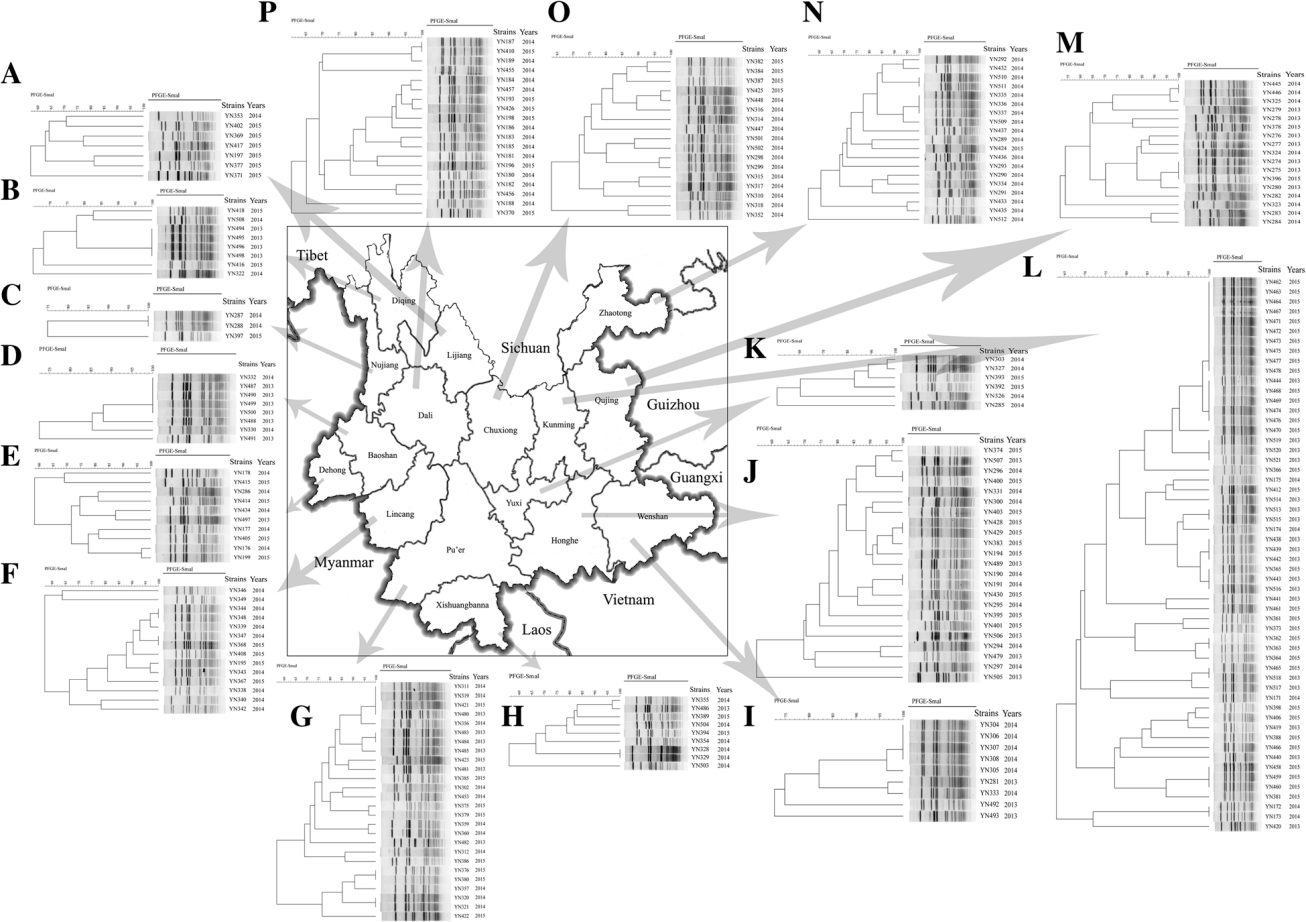
PFGE cluster results of 251 *S.aureus* in southwest China. **a**: Lijiang; **b**: Diqing; **c**: Nujiang; **d**: Baoshan; **e**: Dehong; **f**: Lincang; **g**: Pu’er; **h**: Xishuangbanna; **i**: Wenshan; **j**: Honghe; **k**: Yuxi; **l**: Kunming; **m**: Qujing; **n**: Zhaotong; **o**: Chuxiong; **p**: Dali

PFGE examination revealed 163 PFGE-*SmaI* patterns among all the *S. aureus* isolates. The isolates showed great heterogenous PFGE patterns distribution, especially for Lijiang, Nujiang, Dehong, Lincang, Pu’er, Xishuangbanna, Honghe, Yuxi, Dali, Chuxiong, Zhaotong and Qujing cities, almost no identical PFGE patterns were shown in these cities (Fig. 2a, c, e, f, g, h, j, k, m, n, o and p). However, some strains showed identical patterns, such as YN494, YN495, YN496 and YN498 in Diqing (Fig. 2b); YN332, YN487, YN490, YN499 and YN500 in Baoshan (Fig. 2d.); YN304, YN306, YN307 and YN308 in Wenshan (Fig. 2i); YN462, YN463, YN464, YN467, YN471, YN472, YN473, YN474, YN477 and YN478 in Kunming (Fig. 2l). Specifically, most of these strains were isolated from rice noodles and rice rolls.

### MLST

**Fig. 3:**
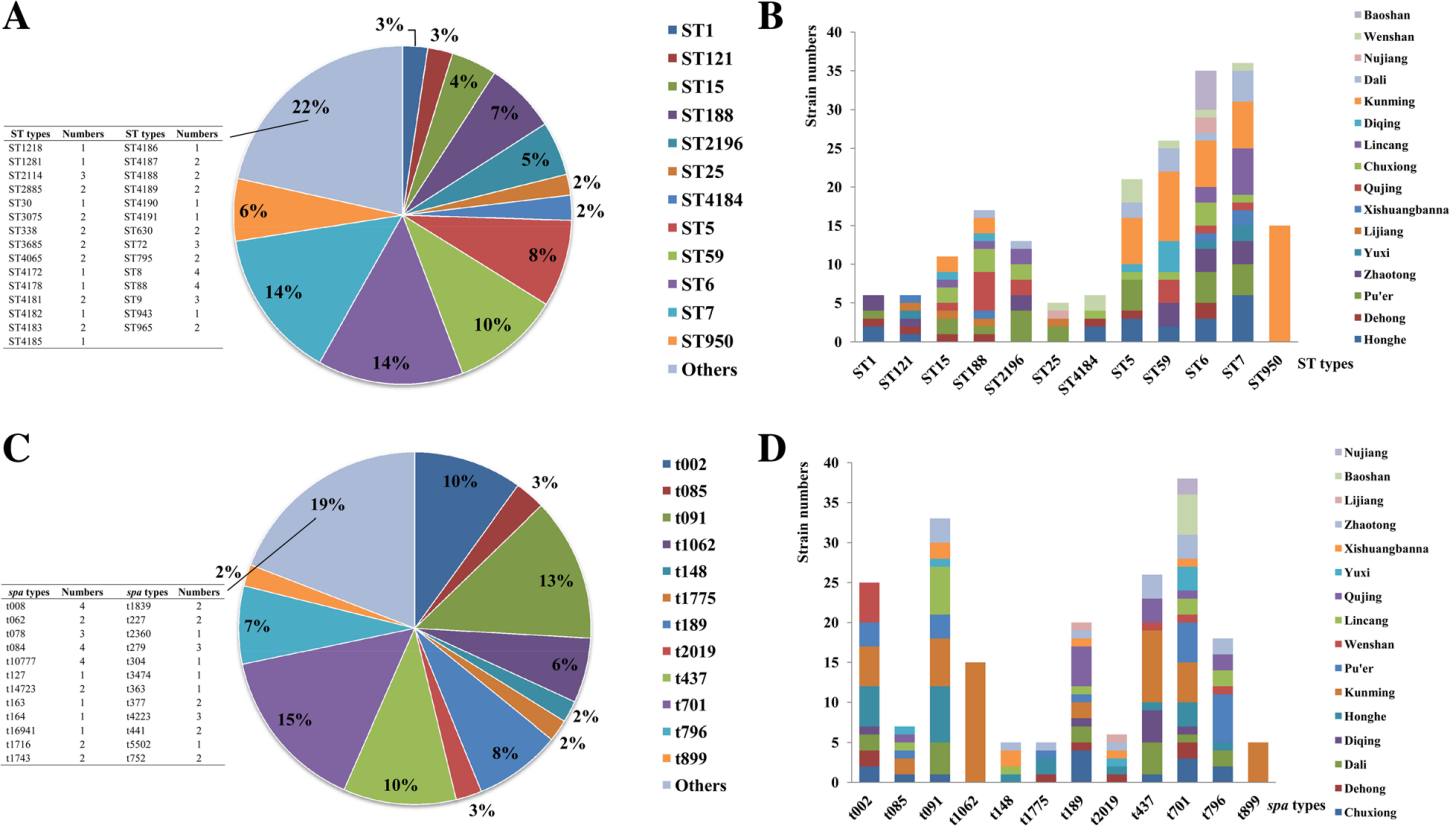
MLST and *spa* typing results of *S. aureus* in this study. **a**: The ST types distributions; **b**: The ST types distribution in 16 cities; **c**: The *spa* types distributions; **d**: The s*pa* types distribution in 16 cities

A total of 41 ST types were obtained; among them ST4172, ST4178, ST4181 to ST4191 were isolated for the first time in China (Additional file 1). ST7 (36/251, 14%), ST6 (35/251, 14%), ST59 (26/251, 10%) and ST5 (21/251, 8%) were the most common ST types distributed in Yunnan province, accounted for 46% of all the *S. aureus* (Fig. 3a). The majority of the ST types were discrete distributed in 16 cities, and each city had different diversified ST types, except the ST950 in Kunming (Fig. 3b).

### *Spa* typing

A total of 36 *spa* types were obtained for the 251 *S. aureus* isolates (Additional file 1); t701 (38/251, 15%), t091 (33/251, 13%), t437 (26/251, 10%) and t002 (25/251, 10%) were the prevalent *spa* types, accounted for 48% (Fig. 3c). The *spa* types of isolates also discrete distributed in 16 cities, and each city had different diversified *spa* types, except t1062 and t899 in Kunming (Fig. 3d).

**Fig. 4:**
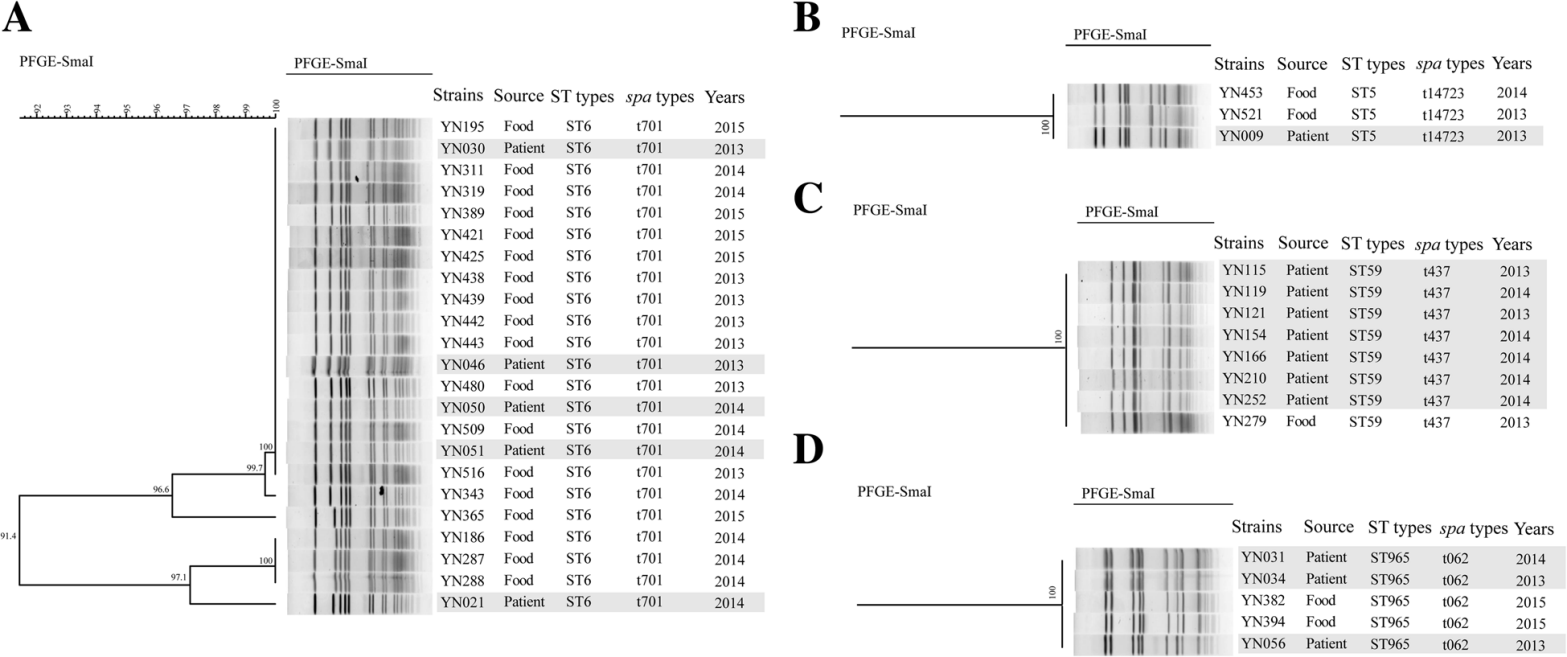
PFGE cluster results for patients strains with food strains. **a**: ST6-t701 type; **b**: ST5-t14723 type; **c**: ST59-t437 type; **d**: ST965-t062 type. The grey areas of figure indicated the *S. aureus* isolated from patients

Together analysis MLST with *spa* typing, ST6-t701 (33/251, 13.15%), ST7-t091 (32/251, 12.75%), ST59-t437 (25/251, 9.96%) and ST5-t002 (19/251, 7.57%) were the most types for all the isolates, accounted for 43.43%. These strains were discrete distributed in different cities, no dominant type was found. The comparison of *S. aureus* isolates from patients and food was also performed; the results showed that some strains from patients had identical PFGE patterns, ST and *spa* types with food isolates, indicated high homology relationship. These strains were specifically for ST6-t701 (Fig. 4a), ST5-t14723 (Fig. 4b), ST59-t437 (Fig. 4c) and ST965-t062 (Fig. 4d). Among them, ST6-t701 was the prevalent type, since five strains of patients from our database showed identical patterns with food strains and the majority of food strains were isolated from rice noodles and rice rolls.

### SCC*mec* typing of MRSA

SCC*mec* typing was performed on 74 MRSA isolates in this study. Among them, type IV and V were found between all these strains. 74.32% of all MRSA were type IV (55/74), 20.27% were type V (15/74), and four isolates could not be assigned (Table 2). Combined analysis of MLST, *spa* and SCC*mec* types for MRSA indicated ST6-SCC*mec* IV-t701 (36.50%) was the most prevalent clone among MRSA isolates from food, followed by ST59-SCC*mec* V-t437 (20.30%), ST5-SCC*mec* IV-t002 (12.20%) and ST59-SCC*mec* IV-t437 (12.20%).

**Table 2:** Molecular types of MRSA isolates from food in this study

MLST	*spa* type	SCC*mec*			Numbers
		IV	V	NA	
ST1	t1775	4(5.40%)	–	–	4
ST5	t002	9(12.20%)	–	–	9
ST6	t701	27(36.50%)	–	–	27
ST7	t091	4(5.40%)	–	4(5.40%)	8
ST59	t437	9(12.20%)	15(20.30%)	–	24
ST88	t10777	2(2.60%)	–	–	2

## Discussion

SEs produced by toxigenic strains are predisposing causes of SFP, among them, SEA is the most common ones. In this study, *sea*, *seb*, *sec* and *sed* were the most found toxin genes, 14.34% of the strains had *tst*, and 16.73% had *PVL*. Some similar results could be found from other Chinese studies, for example, Chao et al. [15] analyzed 568 isolates from different sources for the prevalence of SEs genes and performed *spa* typing. Approximately 54.40% of the isolates from different sources harbored one or more SE genes. Seven genes, *sea*, *seb*, *seg*, *seo*, *sem*, *seq*, and *sel* were more frequently detected. In Li et al. study, [16] the most detected toxin genes were *seu*, *lukED*, *hla*, *hlb* and *hld*, followed by *hlg*, *hlgv*, *lukPV*, *sea*, *see*, *etd*, *seb*, *sec*, *sed*, *sej*, and *sek*. Although *S. aureus* strains in this study were isolated from food surveillance and not from SFP outbreak or patients, the high carriage rates of virulence genes indicated high risk for SFP in case of improper food handling and compromised good hygienic practices during food production and storage.

Several studies showed that high levels of MRSA were found on US and European farms and commercially-distributed meats [17–19]. The prevalence of MRSA isolated from raw retail meat products was reported, ranging from less than 1% in Asia [20] to 11.9% in the Netherlands [21], with intermediate prevalence in other studies [22, 23]. A recent study found that 45% of pork and 63% of beef products were positive for *S. aureus* in the United States [24]. However, other studies reported low isolation rates of MRSA in foods. El-Bayomi et al. [25] analyzed 110 samples from chicken products, and 30 samples were positive for *S. aureus*. Examination of virulence-associated factors revealed that 31.3% of isolates harbored the *PVL* gene, whereas 10.4% were positive for the *sea* and *sed* genes each. Thapaliya et al. [26] investigated *S. aureus* and MRSA in commercially-distributed raw meat products. The prevalence of *S. aureus* on meat samples was 27.8%. Higher prevalence of MRSA was found in meat samples. Among the *S. aureus* isolates, 18 were *PVL*-positive and 41 for the *mecA* gene. Al-Ashmawy et al. [27] analyzed the MRSA isolated from raw milk and dairy products in Egypt, and the average isolation rate of MRSA was 53% among all milk and dairy products, with 35, 50, 40, 65 and 75% in yogurt samples, ice cream, Kareish cheese, Damietta cheese, and raw milk, respectively. In our study, 29.50% of isolates were MRSA, being a higher isolation rate to the reported in the above study. ST6-SCC*mec* IV-t701 was the most prevalent clone among MRSA isolates from food, followed by ST59-SCC*mec* V-t437, ST5-SCC*mec* IV-t002 and ST59-SCC*mec* IV-t437. Most of these strains were from grains and meat products. Grains are the favorite food for Chinese population; it can be processed into a variety of products, such as steamed bread, noodles, bean jelly, rice noodles and rice rolls etc. Among them, rice noodles, rice rolls are the most favorite foods in southwest China, people lived there eat these foods almost every day, and they eat them raw and cold. The comparison between food and patients’ isolates, where a high homology was observed for ST6-t701 types isolated from rice noodles and rice rolls, indicated the possible circulation of those types between patients and foods. Therefore, the origin and distribution of MRSA or *S. aureus* are different between countries probably due to their specific eating habits and the resources of food.

Some studies performed in Europe indicated that ST398 was the most common MRSA type in retail meat, which might be a potential transmission factor of ST398 from farm to the community [28]. Yan et al. [29] analyzed 52 *S. aureus* isolates from 11 outbreaks in Shenzhen, China. They found that ST6 was the major ST type, and constituted 63.5% of all the strains in seven outbreaks. The second most commonly found ST type was ST943, constituted 23.1% of all the isolates from three outbreaks. The dominant *spa* types were t701, t091 and t2360, accounting 67.3% of the isolates. Li et al. [16] investigated the molecular epidemiology of *S. aureus* isolates from seven SFP outbreaks between 2006 and 2013 in Xi’an, northwest China. The results showed that ST6-t701 (71.4%) was the dominant clone, followed by ST5-t002 (14.3%) and ST59-t172 (14.3%). The antibiotic resistance results showed that 71.4% of the strains were resistant to trimethoprim and penicillin, 28.6% resistant to erythromycin, and 14.3% to ampicillin, clindamycin, and tetracycline. All the isolates were sensitive to chloramphenicol, cefoxitin, oxacillin, amikacin, and vancomycin. Furthermore, studies referred to MRSA circulated in the China hospitals revealed that ST59-SCC*mec* IV-t437 was the most common epidemic clone [30, 31]. All these researches mentioned above indicated that ST6-SCC*mec* IV-t701 and ST59-SCC*mec* IV-t437 were predominant MRSA genotypes for patients. In this study, ST6-t701, ST7-t091, ST59-t437 and ST5-t002 were the prevalent types of the isolates, accounted for 43.43%. ST6-SCC*mec* IV-t701, ST59-SCC*mec* V-t437, ST5-SCC*mec* IV-t002 and ST59-SCC*mec* IV-t437 were the most prevalence MRSA clones. The strains showed great heterogenous distributions characteristics; however, ST6-t701 (or ST6-SCC*mec* IV-t701) was the dominant type in southwest China, because of their homology with strains isolated from patients and the aforementioned outbreaks.

This study was the first systemic *S. aureus* food surveillance in southwest China, in which the molecular characteristics of strains were determined. However, there were several limitations in this study. Firstly, the study was conducted in urban regions that probably showed a poor representation of the potential *S. aureus* food surveillance. Secondly, the surveillance time was only for three years, which might not fully reflect the characteristics of local strains. Therefore, further research including urban, rural, and other food sources might be done to comprehensively evaluate the prevalence and features of *S. aureus*.

## Conclusions

Two hundred fifty-one *S. aureus* for food surveillance were analyzed from 2013 to 2015 in southwest China. Isolation rate of *S. aureus* was 2.60% during the three years’ surveillance and 29.50% of them were MRSA. 163 PFGE-*SmaI* patterns were obtained for all the strains and the isolates displayed great heterogenous distributions. ST6-t701 (13.15%), ST7-t091 (12.75%), ST59-t437 (9.96%) and ST5-t002 (7.57%) were the most types for all the isolates, scattered distributed in different cities. ST6-SCC*mec* IV-t701 was the most prevalent clone among MRSA isolates from food, followed by ST59-SCC*mec* V-t437, ST5-SCC*mec* IV-t002 and ST59-SCC*mec* IV-t437. Some strains had the identical PFGE patterns, ST and *spa* types with isolates from patients. *S. aureus* isolated from food in southwest China displayed heterogeneity. Isolates had the same genotype profiles with isolates from patients, indicating high homology. Future research including urban, rural, and other food sources might be done to comprehensively evaluate the prevalence and features of *S. aureus* in southwest China.

## Methods

### Sample collection and isolation of *S. aureus*

The collection of samples used in this study was complied with national regulation on risk monitoring and management of food safety guidelines, and permitted by Center for Food Safety Risk Assessment of Yunnan Province. A total of 9646 food samples were collected from 16 cities in Yunnan province, from 2013 to 2015. The types of foods included grain products, meat products, milk products, fishery products, drinks, pastry, fruit products and vegetable products etc. The food samples were processed based on *S. aureus* isolation methods for foods of national standards in China (GB/T 4789.10–2010). We used qualitative method to isolate the strains. Each sample was aseptically weighed in an analytical balance, twenty-five grams were placed into a sterile plastic bag and 225 mL of buffered peptone water (Oxoid, UK) was added, then homogenized in a Stomacher Bagmixer 400 W (Interscience, France) for one minute. The subsequent enrichments and isolation process were according to the method described by Puah et al. [32]. All the isolated *S. aureus* were identified by Vitek Compact 2 (bioMérieux, France). *S. aureus* isolated from patients from hospital surveillance of Yunnan province were used for comparison to the food isolates.

### Antibiotic resistance test

Minimum inhibitory concentrations (MICs) for 13 antibiotics was determined through broth micro-dilution method using customized microtiter plates (Sensititre, UK) according to the manufacturers’ instructions: Penicillin (PCN), Oxacillin (OXA), Gentamicin (GEN), Ciprofloxacin (CIP), Levofloxacin (LEV), Moxifloxacin (MXF), Erythromycin (EM), Clindamycin (CM), Linezolid (LZD), Vancomycin (VAN), Tetracycline (TET), Rifampicin (RIF) and Trimethoprim/Sulfamethoxazole (SXT). The tests were performed and interpreted in accordance to the Clinical and Laboratory Standards Institute (CLSI) guidelines (M100-S25, 2015); *S. aureus* ATCC 29213 was used as quality control. The *mecA* gene was detected by PCR, as described by Velasco et al. [33].

### Virulence genes detection

The genomic DNA of the bacteria was extracted using Bacterial Genomic Extraction Kit (Tiangen, Beijing) followed the manufacturer’s instructions. The virulence genes were detected based on previous study [34]. The enterotoxin, toxic shock syndrome toxin, exfoliative toxin*,* leucocytic toxin, hemolysin and epidermic cell differential inhibition gene were all amplified (MyCycler, Bio-Rad). Taq premix (TaKaRa, Japan) was used, and the amplification procedures were 94 °C 5 min followed by 30 cycles: 94 °C 15 s, 55 °C 30s, 72 °C 30s, and finally 72 °C 10 min. Electrophoresis of the amplified products was performed in 1.5% agarose, and detected by Gel Imaging instrument (Bio-Rad, USA).

### Pulsed-field gel electrophoresis

PFGE was performed according to Coombs et al. [35], and each plug was digested with 50 U *SmaI* (TaKaRa, Japan) at 37 °C for 4 h. CHEF-Mapper (Bio-Rad, USA) was used for electrophoresis, and the pulse time ranged from 4 s to 40 s for 19 h. The gel was stained with Gel Red (Biotium) and visualized using a gel imaging system (Bio-Rad, Gel DocXR). PFGE patterns were analyzed using BioNumerics version 6.6, and a dendrogram was constructed using the Dice coefficient and un-weighted pair group methods with the arithmetic mean algorithm (UPGMA).

### Multilocus sequence typing

MLST was performed according to MLST database (http://saureus.mlst.net) and Enright et al. method [12]. The amplification products were sent for bidirectional sequencing by TaKaRa, Japan; and the sequences were analyzed using DNAStar and MEGA 4.0 software. All the sequences were submitted to MLST database for identifying their allele and ST assignments.

### *Spa* typing

The genomic DNA of the bacteria was extracted as described above. PCR amplification and sequencing primers were used according to Harmsen et al. method [36]. The amplification products were sent for bidirectional sequencing by TaKaRa, Japan, and the sequences were submitted to the database for their *spa* type assignments (http://spatyper.fortinbras.us/).

### SCC*mec* typing

SCC*mec* typing was performed by discriminating the *mec* complex and the cassette chromosome recombinase (*ccr*) genes complex as described previously [37], which was based on a set of multiplex PCR methods. SCC*mec* types were assigned according to the combination of the *mec* class and *ccr* type. MRSA strains which could not be assigned were defined as non-typable (NA).

### Statistical analysis

Data analysis was performed by IBM SPSS software (version 19.0 for Windows, Armonk, NY). Non-parameter analysis was performed using a Kruskal-Wallis H test in SPSS statistics package. Two-sided significance was assessed for all variables, applying a cut-off value of *P*<0.05.

## Additional file

Additional file 1: The bacterial information, antibiotic resistance and molecular typing results for all the strains used in this study. (XLS 121 kb)

## Abbreviations

MLST: Multilocus sequence typing
MRSA: Methicillin-resistant *S. aureus*
PFGE: Pulsed field gel electrophoresis
SEs: Enterotoxins
SFP: Staphylococcal food poisoning
